# Evaluating the efficacy of mesenchymal stem cells for diabetic neuropathy: A systematic review and meta-analysis of preclinical studies

**DOI:** 10.3389/fbioe.2024.1349050

**Published:** 2024-05-06

**Authors:** Yu Li, Guangren Yue, Shuying Yu, Xinhao Cheng, Yilin Cao, Ximei Wang

**Affiliations:** ^1^ Department of Plastic and Reconstructive Surgery, The First Affiliated Hospital of Zhengzhou University, Zhengzhou, China; ^2^ Shanghai Key Laboratory of Tissue Engineering, National Tissue Engineering Center of China, Department of Plastic and Reconstructive Surgery, Shanghai 9th People’s Hospital, Shanghai Jiao Tong University School of Medicine, Shanghai, China

**Keywords:** diabetic neuropathy, neurological disorder, stem cell therapy, mesenchymal stem cells, meta-analysis

## Abstract

Diabetic neuropathy affects nearly half of all diabetics and poses a significant threat to public health. Recent preclinical studies suggest that mesenchymal stem cells (MSCs) may represent a promising solution for the treatment of diabetic neuropathy. However, an objective assessment of the preclinical effectiveness of MSCs is still pending. We conducted a comprehensive search of PubMed, Web of Science, Embase, and Cochrane library to identify preclinical studies that investigate the effects of MSCs on diabetic neuropathy up until 15 September 2023. Outcome indicators consisted of motor and sensory nerve conduction velocities, intra-epidermal nerve fiber density, sciatic nerve blood flow, capillary-to-muscle fiber ratio, neurotrophic factors, angiogenic factors and inflammatory cytokines. The literature review and meta-analysis were conducted independently by two researchers. 23 studies that met the inclusion criteria were included in this system review for qualitative and quantitative analysis. Pooled analyses indicated that MSCs exhibited an evident benefit in diabetic neuropathy in terms of motor (SMD = 2.16, 95% CI: 1.71–2.61) and sensory nerve conduction velocities (SMD = 2.93, 95% CI: 1.78–4.07), intra-epidermal nerve fiber density (SMD = 3.17, 95% CI: 2.28–4.07), sciatic nerve blood flow (SMD = 2.02, 95% CI: 1.37–2.66), and capillary-to-muscle fiber ratio (SMD = 2.28, 95% CI: 1.55 to 3.01, *p* < 0.00001). Furthermore, after MSC therapy, the expressions of neurotrophic and angiogenic factors increased significantly in most studies, while the levels of inflammatory cytokines were significantly reduced. The relevance of this review relies on the fact that summarizes an extensive body of work entailing substantial preclinical evidence that supports the efficacy of MSCs in mitigating diabetic neuropathy. While MSCs emerge as a promising potential treatment for diabetic neuropathy, further research is essential to elucidate the underlying mechanisms and the best administration strategy for MSCs.

## 1 Introduction

According to the International Diabetes Federation, diabetes mellitus currently affects approximately 425 million people worldwide, with projections suggesting that the number could exceed 700 million by 2045 (Sun et al., 2022). Diabetic neuropathy is an early-developing complication of diabetes characterized by axonal degeneration, demyelination, and impaired nerve regeneration. Rest pain, hyperalgesia, and diminished sensation due to diabetic neuropathy affect approximately half of all diabetics, not only dramatically reducing their quality of life but also placing a significant burden on the healthcare system (Pacifico et al., 2023). Although rigorous glucose control can slow the progression of diabetic neuropathy, it is not effective in restoring the function of damaged nerves (Ismail, 2023). Therefore, innovative strategies to improve nerve function in diabetic neuropathy are urgently needed.

Cell-based therapies are considered a promising approach to disease modification due to their ability to harness the body’s healing mechanisms. In particular, mesenchymal stem cells (MSCs), a group of non-hematopoietic stem cells derived from the mesoderm, have attracted considerable attention because of their low immunogenicity, remarkable tissue regeneration potential, and powerful immunomodulatory capacity (Ebrahimi et al., 2023). Furthermore, MSCs are readily available, as they can be isolated from various tissues, including but not limited to bone marrow, adipose tissue, and teeth; they are able to multiply rapidly *in vitro*, undergoing several to a dozen passages (Li and Wang, 2022). Importantly, clinical trials from different countries have demonstrated the safety of MSCs, as evidenced by the fact that no allergic or severe adverse reactions were observed up to years after years of transplantation (Arango-Rodríguez et al., 2023; Razak et al., 2023; Xie et al., 2023). Therefore, treatment of MSCs could offer significant potential in treating tissue damage and functional disorders.

The complexity of diabetic neuropathy derives from its multifaceted interplay of nerve damage, impaired blood supply, and chronic inflammation (Eid et al., 2023). The multifunctional nature of MSCs enables them to exert therapeutic effects through multiple mechanisms. Transplanted MSCs can not only directly participate in tissue regeneration by differentiating into various cell types, such as Schwann cells and vascular endothelial cells, but they can also influence neighboring cells through their paracrine activity (Abuarqoub et al., 2020; Wang et al., 2022). It has been well-established that the secretome of MSCs comprises a diverse array of bioactive molecules that are beneficial for nerve repair. These include neurotrophic factors such as brain-derived neurotrophic factor (BDNF), nerve growth factor (NGF), neurotrophin-3 (NT3), and glial cell line-derived neurotrophic factor (GDNF); angiogenic factors such as vascular endothelial growth factor (VEGF), basic fibroblast growth factor (bFGF), and platelet-derived growth factor (PDGF); as well as anti-inflammatory cytokines, particularly interleukin-10 (IL-10) and transforming growth factor-beta (TGF-β) (Trzyna and Banaś-Ząbczyk, 2021; Drobiova et al., 2023). Consistent with *in vitro* studies, preclinical animal studies have also demonstrated that MSC transplantation significantly relieves the symptoms and slows the progression of diabetic neuropathy, as evidenced by improvements in both motor and sensory nerve function, increased limb blood flow to the limbs, and a reduced inflammatory response (Shibata et al., 2008; Kim et al., 2011; Waterman et al., 2012; Guimarães et al., 2013; Himeno et al., 2013; Hata et al., 2015; Xia et al., 2015; Han et al., 2016; Omi et al., 2016; Brini et al., 2017; Datta et al., 2017; Monfrini et al., 2017; Omi et al., 2017; Abdelrahman et al., 2018; Evangelista et al., 2018; Xie et al., 2019; Hata et al., 2020; He et al., 2020; Kanada et al., 2020; Hata et al., 2021; Pan et al., 2022; Yigitturk et al., 2022; Yang et al., 2023).

Since the clinical application of MSCs in the treatment of diabetic neuropathy is at an early stage, a thorough and rigorous systematic review and meta-analysis of preclinical evidence can greatly help scientists and clinicians in designing and conducting clinical trials. To this end, we reviewed animal studies focused on the use of MSCs for the treatment of diabetic peripheral neuropathy (DPN), obtained from major databases, including PubMed, Web of Science, Embase, and Cochrane library. The main characteristics of the included articles were summarized, and the effectiveness of MSC transplantation on nerve function, blood supply, and inflammation was assessed. We hope that this systematic review and meta-analysis will serve as a basis for incorporating MSCs into the clinical treatment of diabetic neuropathy.

## 2 Materials and methods

### 2.1 Protocol and registration

The research protocol was officially registered in the International Prospective Registry of Systematic Reviews, under the specific identifier PROSPERO CRD42023474123. Moreover, our systematic review was conducted in strict accordance with the 2020 Preferred Reporting Items for Systematic Reviews and Meta-Analyses (PRISMA) guidelines (Moher et al., 2010).

### 2.2 Eligibility criteria

Before study inclusion, clear eligibility criteria were established, and framed using the PICOS model, which stands for population, intervention, comparator, outcomes, and study design. Setting these criteria in advance in accordance with established meta-research methods, ensures an impartial search, selection, and evaluation of relevant studies.

#### 2.2.1 Population (P)

The systematic review focused on preclinical studies using animal models. These models either spontaneously developed diabetic neuropathy or were experimentally induced by streptozotocin (STZ) injection or a high-fat diet. Notably, we excluded studies that were conducted exclusively *in vitro* or included only human clinical trials.

#### 2.2.2 Intervention (I)

For consideration in this review, studies involving the administration of MSCs had to be conducted. No restrictions were placed on the source, route, dosage, timing or frequency of MSC administration. Additionally, the MSCs could be utilized as xenogeneic, allogeneic or autologous products.

#### 2.2.3 Comparator (C)

Animals receiving no treatment, placebo, or an alternative therapeutic modality.

#### 2.2.4 Outcome (O)

The primary outcome of this study was motor nerve conduction velocity (MNCV). Secondary outcomes evaluated included sensory nerve conduction velocity (SNCV), intra-epidermal nerve fiber density (IENFD), sciatic nerve blood flow (SNBF), the capillary-to-muscle fiber ratio in skeletal muscles, neurotrophic factors, angiogenic factors and inflammatory cytokines.

#### 2.2.5 Study design (S)

Controlled interventional studies, whether they were randomized, pseudo-randomized, or non-randomized, were incorporated in this system review. However, unpublished gray literature, abstracts, review articles, editorials, commentaries, and letters were deliberately excluded. There were no exclusions based on the publication date.

### 2.3 Literature search strategy

As of 15 September 2023, a comprehensive and systematic search was conducted in several scientific databases, namely, PubMed, Web of Science, Embase and the Cochrane Library. The specific search strategy can be found in Supplementary Table S1. Additionally, an exhaustive manual search of the reference lists of the identified studies was performed to capture all other relevant research sources.

### 2.4 Study selection process

The articles identified through our systematic search were cataloged and managed using EndNote 9 software (Clarivate Analytics, USA). The titles and abstracts were methodically screened by two independent reviewers, who subsequently undertook an in-depth evaluation of the full text for relevant research. When discrepancies arose between the two reviewers, a third team member was consulted to achieve consensus through extensive discussion.

### 2.5 Data extraction

The relevant data from the selected articles were independently extracted by two authors. In cases of discrepancies between them, the differences a third team member was consulted to achieve consensus through extensive discussion. Data collected included: (1) study characteristics such as first author, year of publication, and country of the study; (2) details of the study population, including gender, body weight, method of inducing the diabetes model, and others; (3) intervention specifics such as the source of MSCs, any MSC pre-treatment methods, dosage of MSCs, and the source of administration of MSCs; (4) elements of the study design, including sample size and group comparison; (5) Outcomes, for example, motor nerve conduction velocity, sensory nerve conduction velocity, intra-epidermal nerve fiber density, sciatic nerve blood flow, neurotrophic factors, angiogenic factors and inflammatory cytokines.

### 2.6 Risk of bias

The risk of bias in animal experiments was evaluated independently by two authors, using the Systematic Review Centre for Laboratory Animal Experimentation’s risk of bias (SYRCLE’s ROB) tool (Hooijmans et al., 2014). Selection bias was assessed through sequence generation, baseline characteristics, and allocation concealment. Performance bias was evaluated by implementing random housing and blinding of both participants and personnel. Detection bias was examined by conducting random outcome assessments and ensuring blinding of these assessments. We screened for attrition bias based on incomplete outcome data and reporting bias due to selective reporting. Additionally, other potential sources of bias were systematically evaluated.

### 2.7 Data analysis

Data analyses were carried out utilizing Review Manager 5.3 software (Cochrane Collaboration, UK) in conjunction with StataMP-64 Software (StataCorp LP, USA). All outcomes were considered as continuous data and articulated using the standardized mean difference (SMD) accompanied by 95% confidence intervals (CIs). We set a pre-defined threshold at *p* < 0.05 to delineate statistical significance. Considering variations in assessment time points across the reviewed studies, our approach involved adopting the maximum effect estimates from each study, specifically within a consistent 7- to 14-week interval, to facilitate pooled analyses. To evaluate the heterogeneity among the included studies, we employed the I^2^-statistic test. In instances where I^2^ was 50% or below, indicating minimal or no significant heterogeneity, a fixed-effects model was chosen. Conversely, if I^2^ exceeded 50%, indicating significant heterogeneity, a random-effects model was employed (Higgins and Thompson, 2002).

## 3 Results

### 3.1 Search results

**FIGURE 1:**
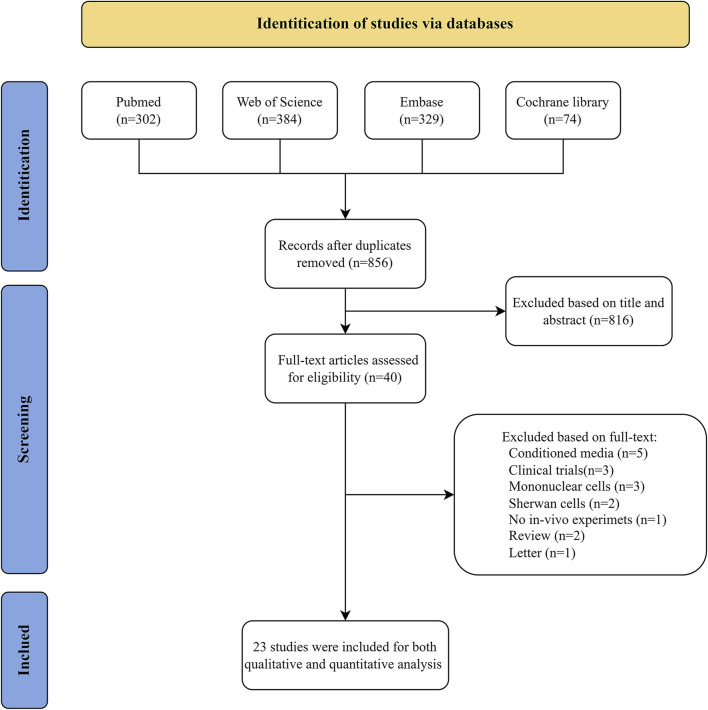
PRISMA flow diagram illustrating the literature search process.

The entire article selection process is summarised in Figure 1. A total of 1,089 studies were identified from the PubMed, Web of Science, Embase and Cochrane Library databases, with 856 records retrieved after removing duplicates. Following the screen of the titles and abstracts, a total of 816 articles were excluded from consideration due to not meeting inclusion criteria. The full text of the remaining 40 studies was examined for eligibility assessment, resulting in 23 records for system review.

### 3.2 Characteristics of the included studies

**FIGURE 2:**
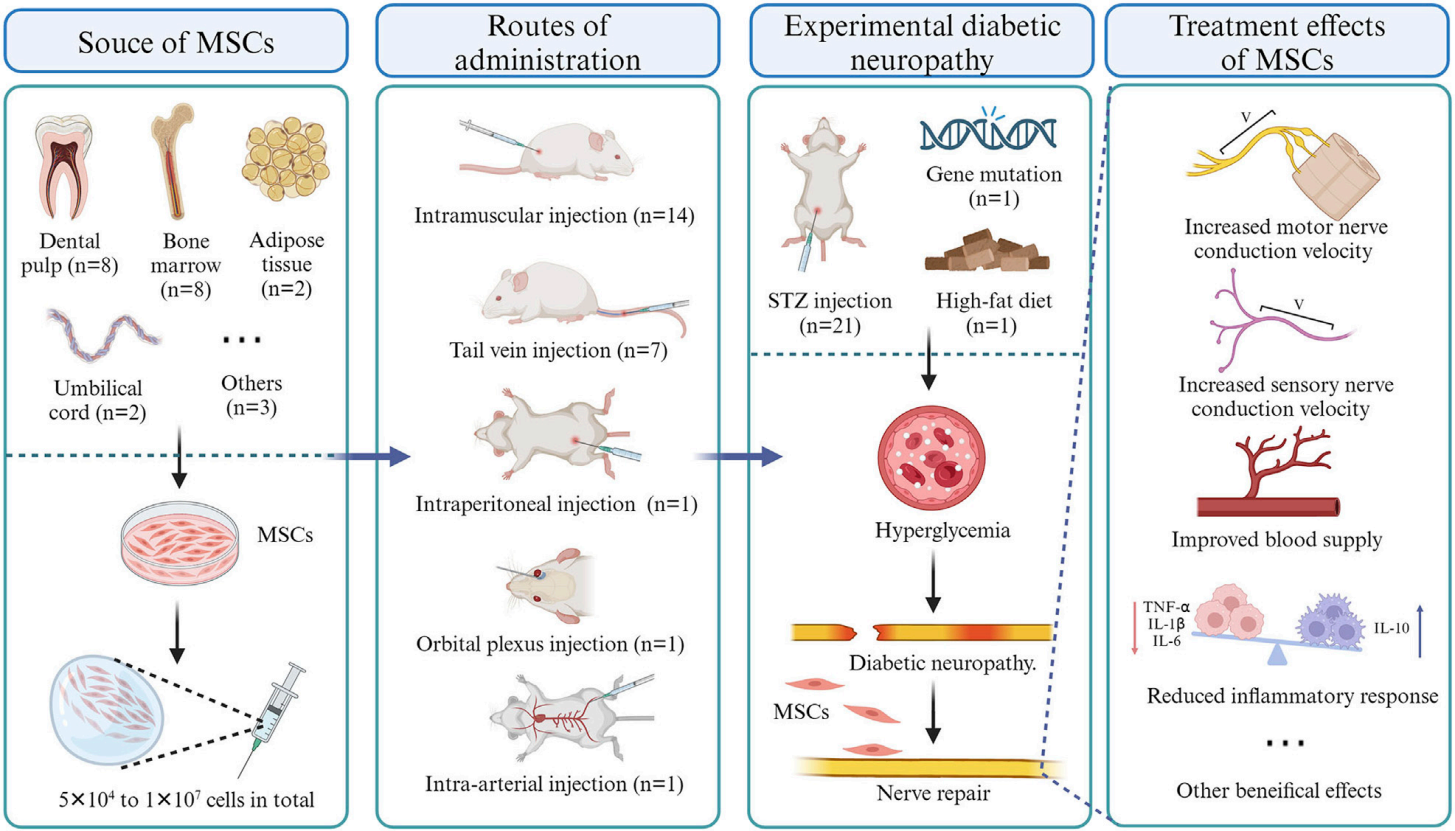
Overview of MSC therapy for experimental diabetic neuropathy.

As of 15 September 2023, a total of 23 eligible studies were included and their salient characteristics are summarized in Table 1 and Figure 2. These studies were sourced from nine different countries and regions, with about a third originating from Japan. Of the 23 included studies, 21 used STZ-induced diabetic rodents (mice and rats), while the other two utilized gene-deficient diabetic rodents with or without a high-fat diet. The strains of included animals encompass Sprague–Dawley rats (n = 7), C57Bl/6 mice (n = 6), BALB/c mice (n = 4), Wistar rats (n = 3), Lewis rats (n = 1), Db/db mice (n = 1), and Goto-Kakizaki rats (n = 1). The dosages of MSCs across studies ranged from 5 × 10^4^ to 1 × 10^7^ cells, with allogeneic cells used in 14 studies and xenogeneic cells in nine studies. These cells were isolated from dental pulp (n = 9), bone marrow (n = 8), umbilical cord (n = 2), adipose tissue (n = 2), placenta tissue (n = 1) or differentiated from pluripotent stem cells (n = 1). of the 23 studies, 13 utilized intramuscular injection only, 6 employed tail vein injection only, 1 utilized arterial injections, 1 employed intraperitoneal injection, 1 utilized orbital plexus injection, and one compared the effectiveness of intramuscular injection *versus* tail vein injection.

**TABLE 1:** The main characteristics of characteristics of all included articles.

Study	Year	Species and number of animals	Sex and Age and Weight	Methods of inducing diabetes	Sources of MSCs	Transplant type	Route of transplantation	Cell dose
Abdelrahman et al	2018	Wistar rats; 20	Male; 12–14 weeks; 180–200 g	STZ (IP)	Rat bone marrow	Allogeneic	IM	5×10^4^; single
Brini et al	2017	C57Bl/6 mice; 12	Male; 9 weeks; 20–25 g	STZ (IP)	Human adipose	Xenogenica	IV	2×10^6^; twice
Datta et al	2017	Wistar rats; 40	Male; 16 weeks; 250–300 g	STZ (IP)	Human dental pulp	Xenogenic	IV or IM	1×10^6^; single or twice
Evangelista et al	2018	C57Bl/6 mice; 12	Male; not reported; 20–25 g	STZ (IP)	Mouse bone marrow	Allogeneic	IV	1×10^6^; single
Guimarães et al	2013	C57Bl/6 mice; 12	Female; 8 weeks; not reported	STZ (IP)	Mouse dental pulp	Allogeneic	OPI	1×10^6^; single
Han et al	2016	Wistar rats; 14	Male; 8 weeks; not reported	STZ (IP)	Rat bone marrow	Allogeneic	IM	5×10^6^; single
Hata et al	2015	Sprague–Dawley rats; 16	Male; 6 weeks; not reported	STZ (IP)	Rat dental pulp	Allogeneic	IM	1×10^6^; single
Hata et al	2020	BALB/cAJcl-nu/nu mice; 8	Male; 6 weeks; not reported	STZ (IP)	Human dental pulp	Xenogenic	IM	1×10^5^; single
Hata et al	2021	BALB/cAJcl-nu/nu mice; 8	Male; 6 weeks; not reported	STZ (IP)	Human dental pulp	Xenogenic	IM	1×10^5^; single
He et al	2020	Sprague–Dawley rats; 32	Male; not reported; 210 g–250 g	STZ (IP)	Rat bone marrow	Allogeneic	IV	1×10^6^, 5×10^6^, or 1×10^7^; single
Himeno et al	2013	C57Bl/6 mice; 15	Male; 5 weeks; not reported	STZ (IP)	Mouse-induced pluripotent stem cells	Allogeneic	IM	1×10^5^; single
Kanada et al	2020	Sprague–Dawley rats; 12	Male; 6 weeks; not reported	STZ (IP)	Rat dental pulp	Allogeneic	IM	1.0×10^6^; single
Kim et al	2011	BALB/c mice; 10	Male; 6 weeks; not reported	STZ (IP)	Mouse bone marrow	Allogeneic	IM	1×10^6^; single
Monfrini et al	2017	Lewis rats; 16	Male; 6 weeks; 175–200 g	STZ (IP)	Rat bone marrow	Allogeneic	IV	1×10^6^; single
Omi et al	2015	Sprague–Dawley rats; 10	Male; 6 weeks; not reported	STZ (IP)	Rat dental pulp	Allogeneic	IM	1×10^6^; single
Omi et al	2017	Sprague–Dawley rats; 10	Male; 6 weeks; not reported	STZ (IP)	Rat dental pulp	Allogeneic	IM	1×10^6^; single
Pan et al	2022	Db/db mice; 27	Male; 9 weeks; not reported	Gene mutation	Human placenta	Xenogenic	IM	1×10^6^; single
Shibata et al	2008	Sprague–Dawley rats; 10	Male; 6 weeks; not reported	STZ (IP)	Rat bone marrow	Allogeneic	IM	1×10^6^; single
Waterman et al	2012	C57Bl/6 mice; 17	Male; 6 weeks; not reported	STZ (IP)	Human bone marrow	Xenogenic	IP	1×10^6^; single
Xia et al	2012	Sprague–Dawley rats; 18	Male; not reported	STZ (IP)	Human umbilical cord blood	Xenogenic	AI	2×10^6^; single
Xie et al	2019	Goto-Kakizaki rats; 20	Male; 10 weeks; 250–300 g	Gene mutation and high-fat feeding	Human exfoliated deciduous teeth	Xenogenic	IV	1×10^7^; twice
Yang et al	2023	C57BL/6 mice; 30	Male; 4–6 weeks; 18 ± 2 g	STZ (IP)	Human umbilical cord	Xenogenic	IV	6×10^4^; three times
Yigitturk et al	2021	BALB/c mice 18	Male; 12 weeks; 20–22 g	STZ (IP)	Mouse adipose	Allogeneic	IM	2×10^6^; single

Number of animals: total number of animals in the control and the MSC-treated groups. AI: arterial injection; IM: intramuscular injection; IP: intraperitoneal injection; IV: intravenous injection; OPI: orbital plexus injection; STZ: streptozotocin.

### 3.3 Quality assessment of the included studies

**FIGURE 3:**
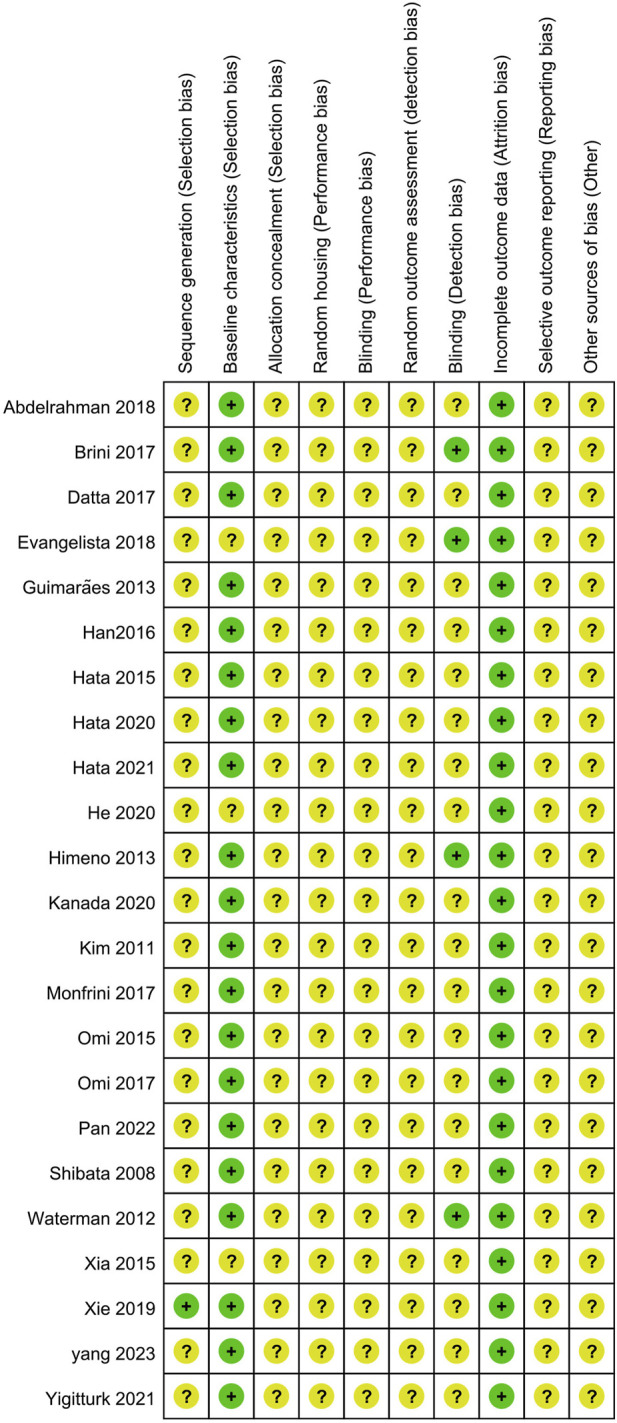
Risk of bias assessment for included studies based on SYRCLE’s ROB tool.

The risk of bias (ROB) among the included studies was assessed using the SYRCLE (Systematic Review Centre for Laboratory Animal Experimentation) tool. However, the majority of domains displayed an unclear ROB. Four studies mentioned the randomization of animal assignments, but only one explicitly reported how they used a random number generator for animal allocation (Xie et al., 2019). Unfortunately, none of the publications explicitly stated the information regarding allocation concealment. 20 studies reported comparable baseline characteristics and were therefore classified as “low risk”. However, three studies were categorized as “unclear risk” due to the omission of age data, making them unevaluable (Xia et al., 2015; Evangelista et al., 2018; He et al., 2020). None of the studies specified whether the allocation was concealed or whether the animals were housed randomly. None of the studies clarified whether experimental caregivers and researchers were blinded to the interventions each animal received. One study reported that all analyses were conducted by personnel unaware of the animals’ identities (Himeno et al., 2013), while three studies indicated that the experimenter was blinded to the treatment group (Waterman et al., 2012; Brini et al., 2017; Evangelista et al., 2018). All studies showed a low attrition bias; however, it remains unclear whether any of the studies selectively reported outcomes (Figure 3). Other potential sources of bias were not reported; hence, we rated all studies as low risk for other sources of bias.

### 3.4 Outcomes of the meta-analysis

#### 3.4.1 Motor nerve conduction velocity (MNCV)

**FIGURE 4:**
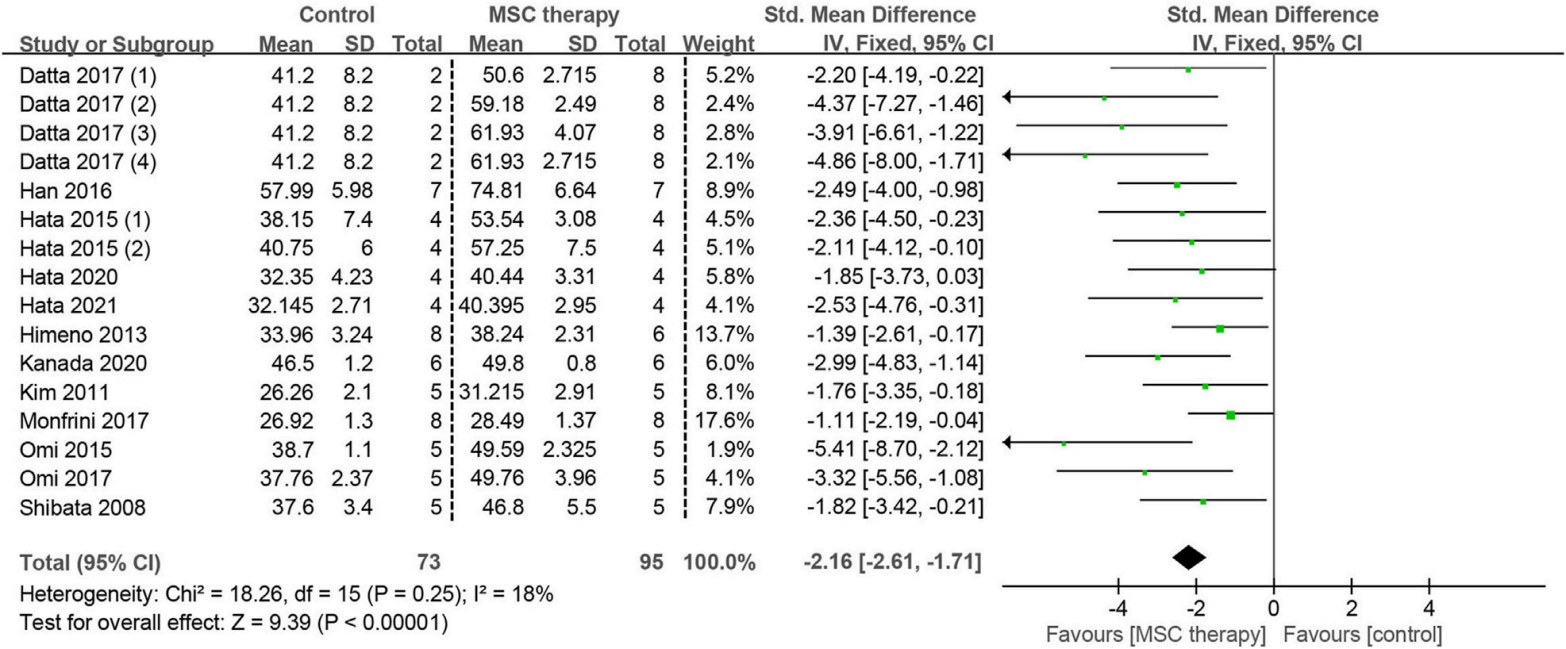
Forest plots illustrating the effects of MSC therapy on MNCV.

Neural conduction velocity is a sensitive and objective indicator of neural function, and its abnormality can suggest the damage of nerve fibres. Twelve studies, involving 168 animals, provided detailed information on MNCV: 11 focused on the sciatic nerve and one on the caudal nerve. As the heterogeneity test revealed low significant heterogeneity (I^2^ = 18%), a fixed-effects model was applied for the quantitative synthesis (Figure 4). A statistically significant improvement in MNCV was observed in the MSC-treated groups compared to the control groups (standardized mean difference [SMD] = 2.16, 95% confidence interval [CI]: 1.71 to 2.61, *p* < 0.00001), suggesting a clear benefit of MSCs on the recovery of motor neural function. Since an adequate number of studies (more than 10) were involved in the quantitative synthesis, the symmetry of the funnel plot was assessed to detect the publication bias. Unfortunately, the funnel plots appeared asymmetrical, suggesting the risk of publication bias in the included studies (Supplementary Figure S1). Consequently, the trim-and-fill method was utilized to evaluate the potential influence of publication bias on the initial conclusion (Supplementary Figure S2). A total of 7 data sets were subjected to the trim and fill procedure, and the recalculated pooled analysis results were similar to the original results (pre-trim-and-fill: SMD = 2.12, CI = 1.68 to 2.57, *p* = 0.0001; post-trim-and-fill: SMD = 5.39, CI = 3.64 to 7.98, *p* < 0.0001). These results provided compelling evidence for the benefits of MSCs on the function of motor nerve fibres.

#### 3.4.2 Sensory nerve conduction velocity (SNCV)

**FIGURE 5:**
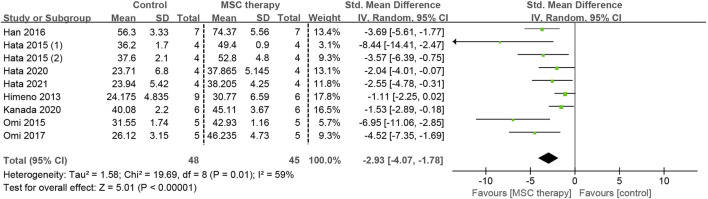
Forest plots illustrating the effects of MSC therapy on SNCV.

The abnormality of sensory nerve function occurs in the early stage of DPN and can be detected before the clinical motor nerve dysfunction. Eight studies, involving 93 animals, provided detailed data on SNCV. Because of the existence of moderate heterogeneity (I^2^ = 59%), a random-effects model was applied for the pooled analysis. The pooled analysis demonstrated that a statistically significant increase in SNCV was observed after the transplantation of MSCs (SMD = 2.93, 95% CI: 1.78 to 4.07, *p* < 0.00001), suggesting that MSCs greatly alleviated the dysfunction of sensory nerve fibers (Figure 5).

#### 3.4.3 Intra-epidermal nerve fiber density (IENFD)

**FIGURE 6:**
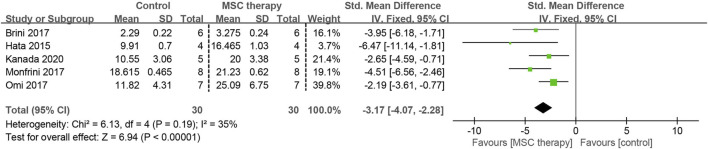
Forest plots illustrating the effects of MSC therapy on IENFD.

Damage to the small diameter nerve fibers within the epidermis is considered an important cause of paresthesia caused by DPN. Five of the included studies, involving 60 animals, investigated the effectiveness of MSCs on IENFD. A fixed-effects model was applied for the meta-analysis due to low heterogeneity among these studies (I^2^ = 35%). All MSC-treated groups exhibited a significantly higher nerve fiber density compared to the control group (SMD = 3.17, 95% CI: 2.28 to 4.07, *p* < 0.00001) (Figure 6).

#### 3.4.4 Sciatic nerve blood flow (SNBF)

**FIGURE 7:**
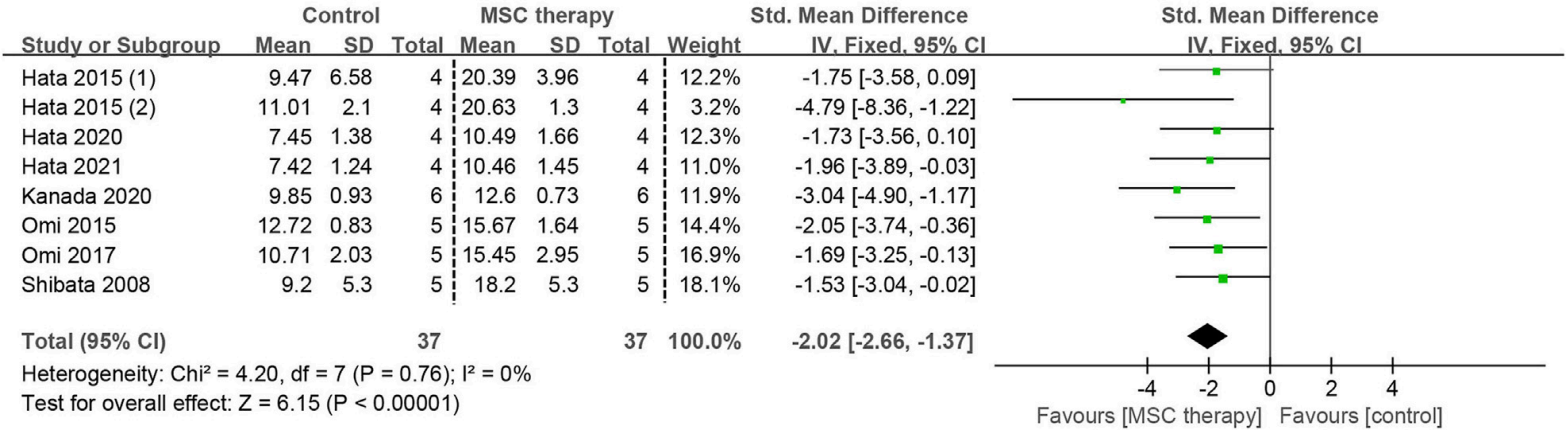
Forest plots illustrating the effects of MSC therapy on SNBF.

Insufficient blood supply is another significant factor contributing to nerve dysfunction in DPN. Detailed information on SNBF was found in 7 studies involving 74 animals. Since there was no heterogeneity among these studies (I^2^ = 0%), a fixed-effects model was applied for the quantitative synthesis. Pooled analysis revealed that MSC-treated groups had significant improvement in sciatic nerve blood flow compared to control groups (SMD = 2.02, 95% CI: 1.37 to 2.66, *p* < 0.00001). This demonstrates an enhanced blood supply to the sciatic nerve as a result of MSC therapy (Figure 7).

#### 3.4.5 Capillary-to-muscle fiber ratio of skeletal muscles

**FIGURE 8:**
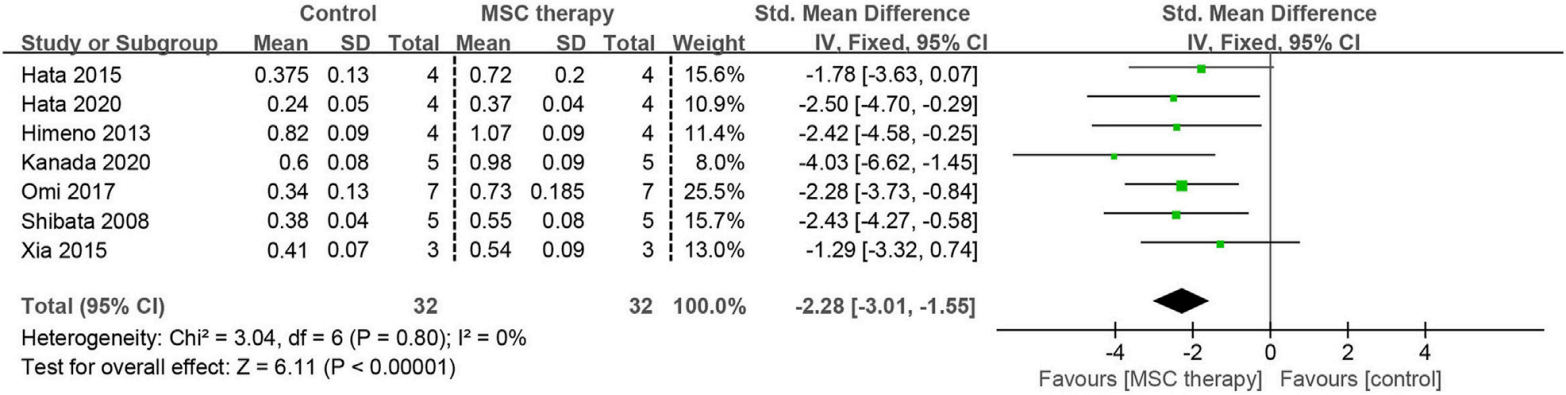
Forest plots illustrating the effects of MSC therapy on the capillary-to-muscle fiber ratio in skeletal muscles.

Microcirculation disorder is one of the primary pathological features of DPN, with the capillary density of skeletal muscles serving as a dependable marker for this process. Data on the capillary-to-muscle fibre ratio were detailed in seven studies involving 64 animals. No data heterogeneity was observed among these studies (I^2^ = 0%), yet a significant association was found between MSC therapy and the increase in the capillary-to-muscle fiber ratio (SMD = 2.28, 95% CI: 1.55 to 3.01, *p* < 0.00001) (Figure 8).

### 3.5 Other indicators

#### 3.5.1 Neurotrophic factors

Nine studies sought to elucidate the benefits of MSCs by measuring the expressions of neurotrophic factors (Table 2). Levels of NGF (n = 9, 118 animals) and NT3 (n = 6, 74 animals) in the sciatic nerves or skeletal muscles were assessed using PCR, ELISA, or immunofluorescence. After MSC therapy, a 0.95- to 3.06-fold increase was observed in NGF expression, while the level of NT3 showed a 1.06- to 1.71-fold increase. These results at least partly explain the mechanism through which MSCs exert their neurotrophic effects.

**TABLE 2:** Effects of MSC therapy on the levels of neurotrophic and angiogenic factors.

Study	Method	Site	NGF		NT3		bFGF		VEGF	
			Control group	MSC therapy	Control group	MSC therapy	Control group	MSC therapy	Control group	MSC therapy
Abdelrahman et al. (2018)	qRT-PCR	Sciatic nerves	N/A	N/A	N/A	N/A	N/A	N/A	0.79 ± 0.12	1.54 ± 0.43*
Han et al. (2016)	qRT-PCR	Sciatic nerves	0.85 ± 0.78	2.6 ± 1.42*	N/A	N/A	1.98 ± 1.63	6.26 ± 3.085*	0.69 ± 0.31	3.02 ± 0.94*
Hata et al. (2015)	qRT-PCR	Hindlimb skeletal muscles	1.085 ± 0.50	1.465 ± 0.84*	0.915 ± 0.35	1.10 ± 0.45*	0.96 ± 0.25	1.645 ± 1.02	1.08 ± 0.42	1.04 ± 0.25
Kanada et al. (2020)	qRT-PCR	Hindlimb skeletal muscles	1.025 ± 0.37	1.08 ± 0.18	0.90 ± 0.31	1.23 ± 0.38	0.85 ± 0.16	0.87 ± 0.25	N/A	N/A
Omi et al. (2017)	qRT-PCR	Hindlimb skeletal muscles	0.45 ± 0.21	1.005 ± 0.3*	0.73 ± 0.30	0.87 ± 0.42	1.25 ± 0.40	1.57 ± 0.69	N/A	N/A
Shibata et al. (2008)	ELISA (pg/mL)	Soleus muscles	499.36 ± 76.44	473.885 ± 86.63*	268.37 ± 51.12	284.345 ± 44.73*	N/A	N/A	N/A	N/A
	qRT-PCR	Thigh muscles	N/A	N/A	N/A	N/A	1.22 ± 0.34	1.99 ± 0.37*	1.025 ± 0.34	1.84 ± 0.37*
Xie et al. (2019)	qRT-PCR	Hindlimb skeletal muscles	0.99 ± 0.02	1.805 ± 0.19*	1.00 ± 0.07	1.57 ± 0.07*	1.00 ± 0.01	1.83 ± 0.13*	1.00 ± 0.01	1.18 ± 0.11
He et al. (2020)	qRT-PCR	Sciatic nerves	0.40 ± 0.06	0.62 ± 0.06*	N/A	N/A	N/A	N/A	N/A	N/A
Kim et al. (2011)	qRT-PCR	Sciatic nerves and muscles	0.57 ± 0.10	0.88 ± 0.18*	0.55 ± 0.09	0.94 ± 0.25*	N/A	N/A	N/A	N/A
Yigitturk et al. (2021)	Immunofluorescent staining	Sciatic nerves	21,500 ± 3,000	52,500 ± 2000*	N/A	N/A	N/A	N/A	N/A	N/A

bFGF: basic fibroblast growth factor; NGF: nerve growth factor; NT3: neurotrophin-3; VEGF: vascular endothelial growth factor. *: *p* < 0.05 compared with the control group.

#### 3.5.2 Angiogenic factors

Seven studies provided detailed information on bFGF (n = 6, 74 animals) or VEGF (n = 5, 70 animals). Data were obtained by PCR from sciatic nerves or skeletal muscles (Table 2). The administration of MSCs resulted in a 1.02- to 3.16-fold increase in bFGF levels and a maximum 4.38-fold increase in VEGF levels. However, the MSC-treated group did not show an obvious change in VEGF expression compared to the control group in the study by Hata et al.

#### 3.5.3 Inflammatory cytokines

The chronic, low-grade inflammatory response plays a crucial role in the onset and progression of DPN, and six studies provided data on inflammation-related factors, including tumor necrosis factor-α (TNF-α), interleukin-1 (IL-1), interleukin-1 (IL-6), and IL-10 (5 studies, 94 animals; 5 studies, 90 animals; 2 studies, 37 animals and 4 studies, 60 animals respectively). These samples were collected from various sites, including sciatic nerves, blood plasma, dorsal root ganglia, and spinal cord, and the levels of inflammation-related factors were examined using ELISA or RT-qPCR (Table 3). Generally, the MSC-treated groups consistently showed lower levels of pro-inflammatory factors, including TNF-α, IL-1, and IL-6, but higher levels of the anti-inflammatory factor IL-10 than the control groups. The expression levels of TNF-α in the MSC therapy groups were 28%–68% of those in the control group, while the expressions of IL-1 and IL-6 were 10%–86% and 8%–57% of the control, respectively. Moreover, a 1.56- to 3.58-fold increase was observed in IL-10 expression after MSC therapy. These results suggested that chronic inflammation was alleviated by MSC therapy.

**TABLE 3:** Summary of changes in the expressions of inflammation-related factors after MSC therapy.

Study	Method	Site	TNF-α		IL-1β		IL-6		IL-10	
			Control group	MSC therapy	Control group	MSC therapy	Control group	MSC therapy	Control group	MSC therapy
Omi et al. (2015)	qRT-PCR	Sciatic nerves	4.2 ± 2.15	2 ± 1.02*	3.07 ± 1.92	2.65 ± 1.46	N/A	N/A	0.77 ± 0.51	1.2 ± 0.45
Datta et al. (2017)	ELISA (pg/mL)	Blood plasma	32.12 ± 11.79	16.06 ± 3.05*	N/A	N/A	N/A	N/A	N/A	N/A
Datta et al. (2017)	ELISA (pg/mL)	Blood plasma	32.12 ± 11.79	21.93 ± 5.69*	N/A	N/A	N/A	N/A	N/A	N/A
Datta et al. (2017)	ELISA (pg/mL)	Blood plasma	32.12 ± 11.79	14.7 ± 0.8*	N/A	N/A	N/A	N/A	N/A	N/A
Datta et al. (2017)	ELISA (pg/mL)	Blood plasma	32.12 ± 11.79	16.1 ± 1.8*	N/A	N/A	N/A	N/A	N/A	N/A
Brini et al. (2017)	ELISA (pg/mL)	Sciatic nerves	160.61 ± 55.68	83.33 ± 7.42*	466.67 ± 163.28	46.67 ± 8.16*	1,133.33 ± 204.14	183.33 ± 163.31*	126.53 ± 40.00	453.06 ± 149.98*
Brini et al. (2017)	ELISA (pg/mL)	Dorsal root ganglia	130.30 ± 63.10	71.21 ± 22.27*	252.7 ± 128.84	50 ± 16.66*	2851.85 ± 680.42	216.67 ± 204.12*	167.35 ± 39.98	359.18 ± 99.99*
Brini et al. (2017)	ELISA (pg/mL)	Spinal cord	137.88 ± 55.68	40.91 ± 48.25*	326.61 ± 43.1	40 ± 4.09*	933.33 ± 204.14	166.67 ± 163.28*	200.00 ± 19.99	465.31 ± 199.95*
Evangelista et al. (2018)	ELISA (pg/mL)	Spinal cord	121.25 ± 15.31	68.125 ± 7.65*	18.8 ± 5.88	5.8 ± 1.96*	N/A	N/A	16.2 ± 4.41	25.8 ± 11.76*
Abdelrahman et al. (2018)	qRT-PCR	Sciatic nerves	N/A	N/A	1.83 ± 0.42	1.52 ± 0.35	N/A	N/A	0.56 ± 0.21	1.36 ± 0.46*
Yang et al. (2023)	ELISA (pg/mL)	Blood serum	641.4 ± 20.7	227.59 ± 10.4*	801.72 ± 13.1	461.21 ± 12.9*	609.52 ± 11.4	349.78 ± 10.38*	N/A	N/A
Yang et al. (2023)	ELISA (pg/mL)	Blood serum	641.4 ± 20.7	179.31 ± 10.2*	801.72 ± 3.1	392.24 ± 17.2*	609.52 ± 11.4	283.98 ± 9.89*	N/A	N/A

TNF-α: Tumor Necrosis Factor-alpha; IL-1β: Interleukin-1, beta; IL-6: Interleukin-6; IL-10: Interleukin-10. *: *p* < 0.05 compared with the control group.

#### 3.5.4 Blood glucose concentration

A total of 10 studies, with 16 comparison groups and 188 animals, provided detailed information on blood glucose concentration (Table 4). In seven comparisons, no difference was found between the MSC-treated groups and the control groups. In the remaining nine comparison groups, blood sugar levels were significantly reduced by 10.5%–36.4%. However, blood glucose levels in these groups still exceeded the diagnostic criteria for diabetes, which is a random blood glucose level of >200 mg/dL. These results suggested that although MSC treatment appears to be somewhat effective in alleviating hyperglycemia, it alone may not be sufficient to reduce blood glucose levels to non-diabetic ranges.

**TABLE 4:** Effects of MSC therapy on blood glucose level.

Study	Glucose level (mg/dL)		Observation Period
	Control group	MSC therapy	
Brini et al. (2017)	504.6 ± 86.3	487.1 ± 54.3	7 (w)
Datta et al. (2017) (1)	398.68 ± 16.76	263.82 ± 16.74*	8 (w)
Datta et al. (2017) (2)	398.68 ± 16.76	272.37 ± 24.18*	8 (w)
Datta et al. (2017) (3)	398.68 ± 16.76	253.95 ± 9.31*	8 (w)
Datta et al. (2017) (4)	398.68 ± 16.76	294.08 ± 13.01*	8 (w)
Guimarães et al. (2013)	561.4 ± 20.3	395.1 ± 25.4*	15 (d)
He et al. (2020) (1)	33.2 ± 2.4	28.6 ± 1.3*	3 (w)
He et al. (2020) (2)	33.2 ± 2.4	26.8 ± 1.5*	3 (w)
He et al. (2020) (3)	33.2 ± 2.4	21.1 ± 2.8*	3 (w)
Himeno et al. (2013)	23.10 ± 1.00	22.3 ± 0.82	3 (w)
Monfrini et al. (2017)	505.78 ± 15.90	489.88 ± 20.23	9 (w)
Kanada et al. (2020)	421.70 ± 177.07	412.60 ± 103.30	4 (w)
Omi et al. (2015)	26.60 ± 2.20	23.8 ± 3.2*	4 (w)
Pan et al. (2022)	29.80 ± 3.60	30.3 ± 5.1	4 (w)
Waterman et al. (2012) (1)	466.60 ± 61.20	495 ± 23.68	40 (d)
Waterman et al. (2012) (2)	466.60 ± 61.20	498.60 ± 37.8	40 (d)

D: days; W: weeks. *: *p* < 0.05 compared with the control group.

## 4 Discussion

To the best of our knowledge, this is the first systematic review and meta-analysis that provides a comprehensive synthesis of the preclinical efficacy of MSCs in the treatment of DPN. Pooled estimates from meta-analyses support the hypothesis that MSCs offer significant therapeutic benefits on neural function (i.e., motor and sensory nerve conduction velocities and intra-epidermal nerve fiber density) and vascularization (i.e., sciatic nerve blood flow and capillary-to-muscle fiber ratio). Additionally, the review revealed that the benefits of MSCs may be associated with an increase in neurotrophic factors and angiogenesis, along with an inhibition of inflammatory reactions. These findings suggest that MSC-based therapy could be a promising strategy for the treatment of DPN.

Among the MSCs sourced from various tissues, dental pulp stem cells (DPSCs) and bone marrow mesenchymal stem cells (BMSCs) are the most favored by researchers, being utilized in about two-thirds of the included studies (DPSCs: n = 8; BMSCs: n = 8). DPSCs can be isolated from fallen deciduous teeth or from teeth extracted for orthodontic reasons (e.g., impacted third molars). The easy accessibility of DPSCs makes them a compelling source for cell therapy (Datta et al., 2017). BMSCs were the first MSCs to be discovered, and their safety and efficacy have been confirmed through extensive clinical applications (Chu et al., 2020). Both DPSCs and BMSCs have shown significant potential for restoring nerve dysfunction; however, direct evidence comparing their therapeutic effects on DPN is lacking. Furthermore, the low immunogenicity of MSCs allows the possibility of cross-species transplantation: rodents in nine studies received MSCs derived from human tissue, but no immune rejection was reported (Waterman et al., 2012; Xia et al., 2015; Brini et al., 2017; Datta et al., 2017; Xie et al., 2019; Hata et al., 2020; Hata et al., 2021; Pan et al., 2022; Yang et al., 2023).

Hyperglycemia, the main feature of diabetes, leads to metabolic disorders, redox status imbalance, and cellular dysfunction, all of which contribute to the fundamental pathological basis of DPN (Kaur et al., 2023). 10 of the 23 included studies provide information on the effects of MSCs on hyperglycemia control. After MSC administration, significant decreases in blood glucose were observed in five studies, but rodents in these treatment arms still met the diagnostic criteria for diabetes. MSCs may exert their beneficial effects both by differentiating into pancreatic islet cells and by secreting an array of growth factors that can rejuvenate pancreatic islet cells, such as insulin-like growth factor 1 (IGF-1), pancreatic and duodenal homeobox 1 (PDX-1), and glucagon-like peptide-1 (GLP-1) (Sionov and Ahdut-HaCohen, 2023). Despite these outcomes, MSCs were unable to normalize the blood glucose levels in diabetic rodents, suggesting that their ability to regulate blood glucose may be not as robust as their neurotrophic and angiogenesis functions. This finding suggests that the therapeutic effect of MSCs could be further enhanced by the simultaneous use of hypoglycemic agents.

DPN usually starts with abnormal sensations in the extremities, often accompanied by gradually worsening pain (Pacifico et al., 2023). In later stages, motor nerve involvement may occur, manifesting as decreased muscle tone and strength, ultimately leading to muscle atrophy and paralysis. The conduction velocity of motor and sensory nerves is a sensitive indicator of the condition of limb nerves, and its abnormality often occurs before the clinical manifestations of DPN. A Meta-analysis of 16 treatment arms from 12 included studies showed that MSC administration significantly alleviated delayed motor nerve conduction. The findings were supported by the pooled estimates of sensory nerve conduction from 9 treatment arms in 8 studies. Furthermore, MSC transplantation significantly increased the density of epidermal nerve fibers, suggesting an improvement in the degeneration of terminal nerves. Several studies attributed the benefits of MSCs to their ability to upregulate the levels of neurotrophic factors, such as bFGF and NGF (Han et al., 2016; Hata et al., 2020; Kanada et al., 2020). It is believed that the increase in neurotrophic factors originates not only from the MSCs but also from the cells rejuvenated by them (Yue et al., 2016).

In addition to nerve damage, microangiopathy is also a characteristic pathological feature of DPN. This pathological process is associated with endothelial dysfunction, thickening of the capillary endothelial basement membrane, microcirculatory disorders, and impaired angiogenesis (Fang et al., 2018). Nerve cells rely highly on oxygen and energy; consequently, ischemia resulting from diabetic microangiopathy causes them significant harm. As the longest and thickest peripheral nerve, the sciatic nerve is often the first and most severely affected nerve by diabetic microangiopathy (Kender et al., 2023). Seven studies reported an increase in sciatic nerve blood flow after the administration of MSCs, with a meta-analysis of eight treatment arms showing no significant heterogeneity. The results indicated that the increased blood supply to the sciatic nerve plays a crucial role in the beneficial effects of MSCs on nerve conduction velocity and nerve dysfunction. Furthermore, pooled estimates from seven studies showed an increased ratio of blood vessels to muscle fibers in MSC-treated animals, suggesting an improvement in microcirculation disorder due to MSC treatment.

The inflammatory response helps eliminate pathogens and foreign bodies, and can also activate the body’s natural repair mechanisms. However, when this inflammation becomes chronic and uncontrolled due to diabetes, it can cause considerable damage to the nervous and circulatory systems (Cheng et al., 2024). The immunoregulatory ability of MSCs is also a key factor in their role in neuroprotection and tissue regeneration. It has been reported that the secretome of MSCs contains an abundance of anti-inflammatory factors, such as PGE2, IL-10, IL-35, and TGF-β (Trzyna and Banaś-Ząbczyk, 2021). Furthermore, recent preclinical evidence has revealed that the administration of MSCs can convert a significant proportion of macrophages from a pro-inflammatory phenotype (M1) to an anti-inflammatory phenotype (M2) (Liu et al., 2022). Indeed, several included studies reported a decrease in pro-inflammatory cytokines, including TNF-α and IL-1, and an increase in the anti-inflammatory factor IL-10 in the peripheral nerves, spinal cord and plasma (Omi et al., 2016; Brini et al., 2017; Datta et al., 2017). However, researchers used different methods when assessing inflammation in different tissues, including PCR, ELISA, Western blotting, and immunohistochemistry. This methodological heterogeneity made a meta-analysis not feasible.

We must acknowledge several limitations in this systematic review and meta-analysis. First, all included studies used rodents as animal models, which may exaggerate the treatment effects of MSCs on DPN. Second, although most meta-analysis results showed low heterogeneity, the sample size in each case was limited; Studies with larger samples are urgently needed to provide more robust evidence. Third, the data extracted using the GetData software may have slight differences from the original data.

Overall, MSC therapy showed significant benefits in both neuroprotection and angiogenesis, as evidenced by a number of improvements in MSCs-treated animals compared with the control animals, including enhanced conduction velocity of motor and sensory nerves, denser epidermal nerve fibers, increased sciatic nerve blood flow, and a higher ratio of blood vessels to muscle fibers. The treatment effects of MSCs could also be related to their anti-inflammatory function, but a meta-analysis was not feasible due to the significant methodological differences across studies. These findings suggest that MSC transplantation holds promise as a strategy for DPN. However, rigorous and well-designed preclinical and clinical trials with larger sample sizes remain essential.

## 5 Conclusion

This systematic review and meta-analysis evaluated the preclinical efficacy of MSCs in the treatment of diabetic neuropathy. A comprehensive analysis of 23 animal studies suggests that MSC therapy holds significant potential to improve various aspects of diabetic neuropathy. Pooled analyses indicated that MSC administration resulted in improvements in motor and sensory nerve conduction velocities, increased intra-epidermal nerve fiber density, improved sciatic nerve blood flow, and favorable modulation of the capillary-to-muscle fiber ratio. Additionally, MSC treatment was associated with the upregulation of neurotrophic factors, an increase in angiogenic factors, and a reduction in inflammatory cytokines. While MSCs emerge as a promising avenue for treating diabetic neuropathy based on preclinical models, further research, particularly well-designed clinical trials, is essential to fully understand their therapeutic potential and facilitate their integration into clinical practice.

## Data Availability

The original contributions presented in the study are included in the article/Supplementary Material, further inquiries can be directed to the corresponding author.

## References

[B1] Abdelrahman S. A. Samak M. A. Shalaby S. M. (2018). Fluoxetine pretreatment enhances neurogenic, angiogenic and immunomodulatory effects of MSCs on experimentally induced diabetic neuropathy. Cell Tissue Res. 374 (1), 83–97. 10.1007/s00441-018-2838-6 29687216

[B2] Abuarqoub D. Aslam N. Almajali B. Shajrawi L. Jafar H. Awidi A. (2020). Neuro-regenerative potential of dental stem cells: a concise review. Cell Tissue Res. 382 (2), 267–279. 10.1007/s00441-020-03255-0 32725424

[B3] Arango-Rodríguez M. L. Mateus L. C. Sossa C. L. Becerra-Bayona S. M. Solarte-David V. A. Ochoa Vera M. E. (2023). A novel therapeutic management for diabetes patients with chronic limb -threatening ischemia: comparison of autologous bone marrow mononuclea r cells versus allogenic Wharton jelly-derived mesenchymal stem cells. Stem Cell Res. Ther. 14 (1), 221. 10.1186/s13287-023-03427-z 37626416 PMC10464344

[B4] Brini A. T. Amodeo G. Ferreira L. M. Milani A. Niada S. Moschetti G. (2017). Therapeutic effect of human adipose-derived stem cells and their secretome in experimental diabetic pain. Sci. Rep. 7 (1), 9904. 10.1038/s41598-017-09487-5 28851944 PMC5575274

[B5] Cheng Y. Chen Y. Li K. Liu S. Pang C. Gao L. (2024). How inflammation dictates diabetic peripheral neuropathy: an enlighten ing review. CNS Neurosci. Ther. 30, e14477. 10.1111/cns.14477 37795833 PMC11017439

[B6] Chu D.-T. Phuong T. N. T. Tien N. L. B. Tran D. K. Thanh V. V. Quang T. L. (2020). An update on the progress of isolation, culture, storage, and clinical application of human bone marrow mesenchymal stem/stromal cells. Int. J. Mol. Sci. 21 (3), 708. 10.3390/ijms21030708 31973182 PMC7037097

[B7] Datta I. Bhadri N. Shahani P. Majumdar D. Sowmithra S. Razdan R. (2017). Functional recovery upon human dental pulp stem cell transplantation in a diabetic neuropathy rat model. Cytotherapy 19 (10), 1208–1224. 10.1016/j.jcyt.2017.07.009 28864291

[B8] Drobiova H. Sindhu S. Ahmad R. Haddad D. Al-Mulla F. Al Madhoun A. (2023). Wharton’s jelly mesenchymal stem cells: a concise review of their secretome and prospective clinical applications. Front. Cell Dev. Biol. 11, 1211217. 10.3389/fcell.2023.1211217 37440921 PMC10333601

[B9] Ebrahimi F. Pirouzmand F. Cosme Pecho R. D. Alwan M. Yassen Mohamed M. Ali M. S. (2023). Application of mesenchymal stem cells in regenerative medicine: a new approach in modern medical science. Biotechnol. Prog. 39, e3374. 10.1002/btpr.3374 37454344

[B10] Eid S. A. Rumora A. E. Beirowski B. Bennett D. L. Hur J. Savelieff M. G. (2023). New perspectives in diabetic neuropathy. Neuron 111 (17), 2623–2641. 10.1016/j.neuron.2023.05.003 37263266 PMC10525009

[B11] Evangelista A. F. Vannier-Santos M. A. de Assis Silva G. S. Silva D. N. Juiz P. J. L. Nonaka C. K. V. (2018). Bone marrow-derived mesenchymal stem/stromal cells reverse the sensorial diabetic neuropathy via modulation of spinal neuroinflammatory cascades. J. Neuroinflammation 15 (1), 189. 10.1186/s12974-018-1224-3 29933760 PMC6015468

[B12] Fang F. Wang J. Wang Y. F. Peng Y. D. (2018). Microangiopathy in diabetic polyneuropathy revisited. Eur. Rev. Med. Pharmacol. Sci. 22 (19), 6456–6462. 10.26355/eurrev_201810_16058 30338814

[B13] Guimarães E. T. Da Silva Cruz G. De Almeida T. F. De Freitas Souza B. S. Kaneto C. M. Vasconcelos J. F. (2013). Transplantation of stem cells obtained from murine dental pulp improves pancreatic damage, renal function, and painful diabetic neuropathy in diabetic type 1 mouse model. Cell Transpl. 22 (12), 2345–2354. 10.3727/096368912x657972 23068779

[B14] Han J. W. Choi D. Lee M. Y. Huh Y. H. Yoon Y. S. (2016). Bone marrow-derived mesenchymal stem cells improve diabetic neuropathy by direct modulation of both angiogenesis and myelination in peripheral nerves. Cell Transpl. 25 (2), 313–326. 10.3727/096368915x688209 PMC4889908 25975801

[B15] Hata M. Omi M. Kobayashi Y. Nakamura N. Miyabe M. Ito M. (2020). Transplantation of human dental pulp stem cells ameliorates diabetic polyneuropathy in streptozotocin-induced diabetic nude mice: the role of angiogenic and neurotrophic factors. Stem Cell Res. Ther. 11 (1), 236. 10.1186/s13287-020-01758-9 32546222 PMC7298811

[B16] Hata M. Omi M. Kobayashi Y. Nakamura N. Miyabe M. Ito M. (2021). Sustainable effects of human dental pulp stem cell transplantation on diabetic polyneuropathy in streptozotocine-induced type 1 diabetes model mice. Cells 10 (9), 2473. 10.3390/cells10092473 34572120 PMC8466318

[B17] Hata M. Omi M. Kobayashi Y. Nakamura N. Tosaki T. Miyabe M. (2015). Transplantation of cultured dental pulp stem cells into the skeletal muscles ameliorated diabetic polyneuropathy: therapeutic plausibility of freshly isolated and cryopreserved dental pulp stem cells. Stem Cell Res. Ther. 6 (1), 162. 10.1186/s13287-015-0156-4 26345292 PMC4562193

[B18] He D. Xu Y. Xiong X. Yin C. Lei S. Cheng X. (2020). The bone marrow-derived mesenchymal stem cells (BMSCs) alleviate diabetic peripheral neuropathy induced by STZ via activating GSK-3β/β-catenin signaling pathway. Environ. Toxicol. Pharmacol. 79, 103432. 10.1016/j.etap.2020.103432 32502517

[B19] Higgins J. P. T. Thompson S. G. (2002). Quantifying heterogeneity in a meta-analysis. Statistics Med. 21 (11), 1539–1558. 10.1002/sim.1186 12111919

[B20] Himeno T. Kamiya H. Naruse K. Cheng Z. Ito S. Kondo M. (2013). Mesenchymal stem cell-like cells derived from mouse induced pluripotent stem cells ameliorate diabetic polyneuropathy in mice. Biomed. Res. Int. 2013, 1–12. 10.1155/2013/259187 PMC3844199 24319678

[B21] Hooijmans C. R. Rovers M. M. de Vries R. B. Leenaars M. Ritskes-Hoitinga M. Langendam M. W. (2014). SYRCLE’s risk of bias tool for animal studies. BMC Med. Res. Methodol. 14, 43. 10.1186/1471-2288-14-43 24667063 PMC4230647

[B22] Ismail C. A. N. (2023). Issues and challenges in diabetic neuropathy management: a narrative review. World J. Diabetes 14 (6), 741–757. 10.4239/wjd.v14.i6.741 37383599 PMC10294062

[B23] Kanada S. Makino E. Nakamura N. Miyabe M. Ito M. Hata M. (2020). Direct comparison of therapeutic effects on diabetic polyneuropathy between transplantation of dental pulp stem cells and administration of dental pulp stem cell-secreted factors. Int. J. Mol. Sci. 21 (17), 6064. 10.3390/ijms21176064 32842469 PMC7503871

[B24] Kaur M. Misra S. Swarnkar P. Patel P. Das Kurmi B. Das Gupta G. (2023). Understanding the role of hyperglycemia and the molecular mechanism as sociated with diabetic neuropathy and possible therapeutic strategies. Biochem. Pharmacol. 215, 115723. 10.1016/j.bcp.2023.115723 37536473

[B25] Kender Z. Jende J. M. E. Kurz F. T. Tsilingiris D. Schimpfle L. Sulaj A. (2023). Sciatic nerve fractional anisotropy and neurofilament light chain prot ein are related to sensorimotor deficit of the upper and lower limbs i n patients with type 2 diabetes. Front. Endocrinol. 14, 1046690. 10.3389/fendo.2023.1046690 PMC10053786 37008917

[B26] Kim B. J. Jin H. K. Bae J. S. (2011). Bone marrow-derived mesenchymal stem cells improve the functioning of neurotrophic factors in a mouse model of diabetic neuropathy. Lab. Anim. Res. 27 (2), 171–176. 10.5625/lar.2011.27.2.171 21826178 PMC3146005

[B27] Li Y. Wang X. (2022). Chrysin attenuates high glucose-induced BMSC dysfunction via the activation of the PI3K/AKT/Nrf2 signaling pathway. Drug Des. Devel Ther. 16, 165–182. 10.2147/dddt.s335024 PMC8763623 35058687

[B28] Liu C. Xiao K. Xie L. (2022). Advances in the regulation of macrophage polarization by mesenchymal stem cells and implications for ALI/ARDS treatment. Front. Immunol. 13, 928134. 10.3389/fimmu.2022.928134 35880175 PMC9307903

[B29] Moher D. Liberati A. Tetzlaff J. Altman D. G. (2010). Preferred reporting items for systematic reviews and meta-analyses: the PRISMA statement. Int. J. Surg. 8 (5), 336–341. 10.1016/j.ijsu.2010.02.007 20171303

[B30] Monfrini M. Donzelli E. Rodriguez-Menendez V. Ballarini E. Carozzi V. A. Chiorazzi A. (2017). Therapeutic potential of Mesenchymal Stem Cells for the treatment of diabetic peripheral neuropathy. Exp. Neurol. 288, 75–84. 10.1016/j.expneurol.2016.11.006 27851902

[B31] Omi M. Hata M. Nakamura N. Miyabe M. Kobayashi Y. Kamiya H. (2016). Transplantation of dental pulp stem cells suppressed inflammation in sciatic nerves by promoting macrophage polarization towards anti-inflammation phenotypes and ameliorated diabetic polyneuropathy. J. Diabetes Investig. 7 (4), 485–496. 10.1111/jdi.12452 PMC4931198 27181261

[B32] Omi M. Hata M. Nakamura N. Miyabe M. Ozawa S. Nukada H. (2017). Transplantation of dental pulp stem cells improves long-term diabetic polyneuropathy together with improvement of nerve morphometrical evaluation. Stem Cell Res. Ther. 8 (1), 279. 10.1186/s13287-017-0729-5 29237486 PMC5729514

[B33] Pacifico P. Coy-Dibley J. S. Miller R. J. Menichella D. M. (2023). Peripheral mechanisms of peripheral neuropathic pain. Front. Mol. Neurosci. 16, 1252442. 10.3389/fnmol.2023.1252442 37781093 PMC10537945

[B34] Pan S. Hada S. S. Liu Y. Hu C. Zhou M. Zheng S. (2022). Human placenta-derived mesenchymal stem cells ameliorate diabetic neuropathy via wnt signaling pathway. Stem Cells Int. 2022, 1–11. 10.1155/2022/6897056 PMC9683984 36440182

[B35] Razak H. R. B. A. Corona K. Totlis T. Chan L. Y. T. Salreta J. F. Sleiman O. (2023). Mesenchymal stem cell implantation provides short-term clinical improv ement and satisfactory cartilage restoration in patients with knee ost eoarthritis but the evidence is limited: a systematic review performed by the early-osteoarthritis group of ESSKA-European knee associates s ection. Knee Surg. sports Traumatol. Arthrosc. 31, 5306–5318. official journal of t he ESSKA. 10.1007/s00167-023-07575-w 37737920 PMC10719133

[B36] Shibata T. Naruse K. Kamiya H. Kozakae M. Kondo M. Yasuda Y. (2008). Transplantation of bone marrow-derived mesenchymal stem cells improves diabetic polyneuropathy in rats. Diabetes 57 (11), 3099–3107. 10.2337/db08-0031 18728233 PMC2570407

[B37] Sionov R. V. Ahdut-HaCohen R. (2023). A supportive role of mesenchymal stem cells on insulin-producing lange rhans islets with a specific emphasis on the secretome. Biomedicines 11 (9), 2558. 10.3390/biomedicines11092558 37761001 PMC10527322

[B38] Sun H. Saeedi P. Karuranga S. Pinkepank M. Ogurtsova K. Duncan B. B. (2022). IDF Diabetes Atlas: global, regional and country-level diabetes prevalence estimates for 2021 and projections for 2045. Diabetes Res. Clin. Pract. 183, 109119. 10.1016/j.diabres.2021.109119 34879977 PMC11057359

[B39] Trzyna A. Banaś-Ząbczyk A. (2021). Adipose-derived stem cells secretome and its potential application in "stem cell-free therapy. Biomolecules 11 (6), 878. 10.3390/biom11060878 34199330 PMC8231996

[B40] Wang Q. Chen F. Y. Ling Z. M. Su W. F. Zhao Y. Y. Chen G. (2022). The effect of Schwann cells/schwann cell-like cells on cell therapy for peripheral neuropathy. Front. Cell Neurosci. 16, 836931. 10.3389/fncel.2022.836931 35350167 PMC8957843

[B41] Waterman R. S. Morgenweck J. Nossaman B. D. Scandurro A. E. Scandurro S. A. Betancourt A. M. (2012). Anti-inflammatory mesenchymal stem cells (MSC2) attenuate symptoms of painful diabetic peripheral neuropathy. Stem Cells Transl. Med. 1 (7), 557–565. 10.5966/sctm.2012-0025 23197860 PMC3659725

[B42] Xia N. Xu J. M. Zhao N. Zhao Q. S. Li M. Cheng Z. F. (2015). Human mesenchymal stem cells improve the neurodegeneration of femoral nerve in a diabetic foot ulceration rats. Neurosci. Lett. 597, 84–89. 10.1016/j.neulet.2015.04.038 25916880

[B43] Xie J. Rao N. Zhai Y. Li J. Zhao Y. Ge L. (2019). Therapeutic effects of stem cells from human exfoliated deciduous teeth on diabetic peripheral neuropathy. Diabetol. Metab. Syndr. 11, 38. 10.1186/s13098-019-0433-y 31131042 PMC6525430

[B44] Xie X. Song Q. Dai C. Cui S. Tang R. Li S. (2023). Clinical safety and efficacy of allogenic human adipose mesenchymal st romal cells-derived exosomes in patients with mild to moderate Alzheim er’s disease: a phase I/II clinical trial. General psychiatry 36 (5), e101143. 10.1136/gpsych-2023-101143 37859748 PMC10582850

[B45] Yang L. F. He J. D. Jiang W. Q. Wang X. D. Yang X. C. Liang Z. (2023). Interferon-gamma treatment of human umbilical cord mesenchymal stem cells can significantly reduce damage associated with diabetic peripheral neuropathy in mice. Curr. Stem Cell Res. Ther. 19. 10.2174/1574888x19666230829155046 37644749

[B46] Yigitturk G. Erbas O. Karabay Yavasoglu N. U. Acikgoz E. Buhur A. Gokhan A. (2022). The neuro-restorative effect of adipose-derived mesenchymal stem cell transplantation on a mouse model of diabetic neuropathy. Neurol. Res. 44 (2), 156–164. 10.1080/01616412.2021.1967679 34410214

[B47] Yue Y. Yang X. Zhang L. Xiao X. Nabar N. R. (2016). Low-intensity pulsed ultrasound upregulates pro-myelination indicators of Schwann cells enhanced by co-culture with adipose-derived stem cel ls. Cell Prolif. 49 (6), 720–728. 10.1111/cpr.12298 27625295 PMC6496622

