# Supplementary figures and images for: Evaluating the efficacy of mesenchymal stem cells for diabetic neuropathy: A systematic review and meta-analysis of preclinical studies
Source: Front Bioeng Biotechnol. 2024 May 6;12:1349050. doi: 10.3389/fbioe.2024.1349050 (PMC11102959; doi:10.3389/fbioe.2024.1349050)

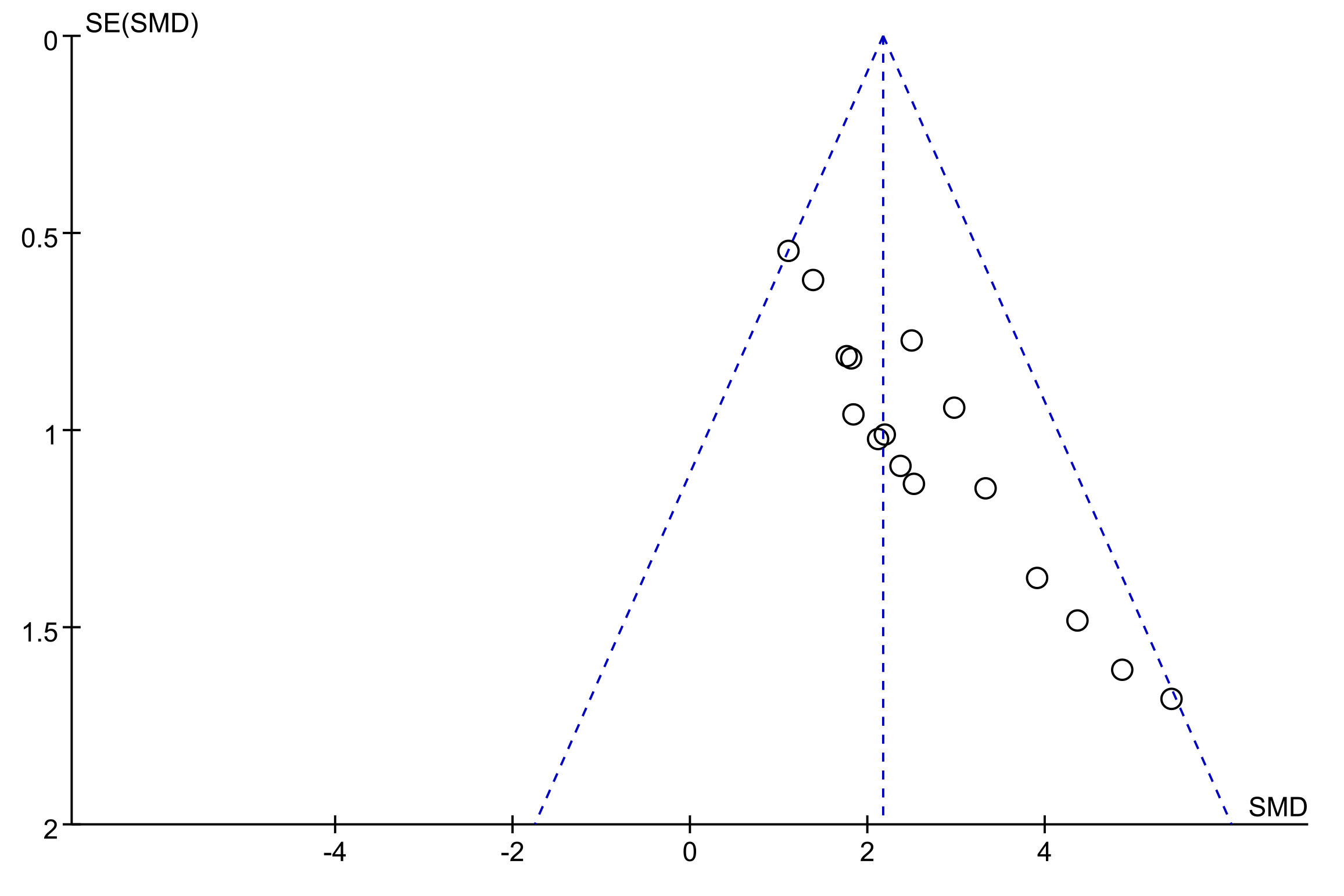

Supplement: Supplementary file 1 [file Image1.JPEG]

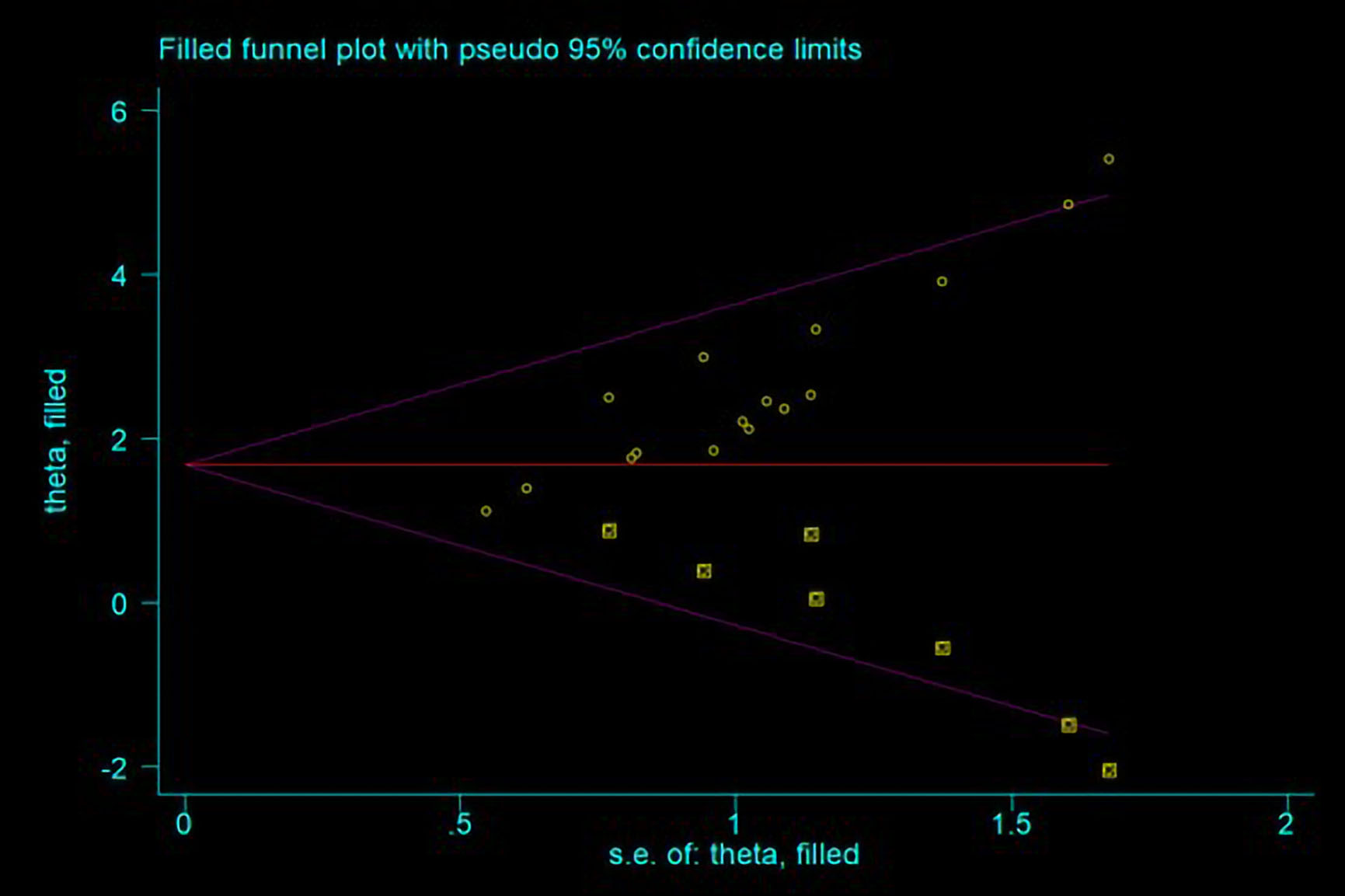

Supplement: Supplementary file 2 [file Image2.JPEG]
