# Supplementary material for: Evaluating the efficacy of mesenchymal stem cells for diabetic neuropathy: A systematic review and meta-analysis of preclinical studies
Source: Front Bioeng Biotechnol. 2024 May 6;12:1349050. doi: 10.3389/fbioe.2024.1349050 (PMC11102959; doi:10.3389/fbioe.2024.1349050)
Supplement: Supplementary file 3 [file DataSheet1.docx]

**Table S1: The specific search strategy for different databases.**

**PubMed:**

#1 Diabetic Neuropathies [MeSH Terms]

#2 (Diabetic Neuropathy[Title/Abstract]) OR (Neuropathies, Diabetic[Title/Abstract]) OR (Neuropathy, Diabetic[Title/Abstract]) OR (Diabetic Autonomic Neuropathy[Title/Abstract]) OR (Autonomic Neuropathies, Diabetic[Title/Abstract]) OR (Autonomic Neuropathy, Diabetic[Title/Abstract]) OR (Diabetic Autonomic Neuropathies[Title/Abstract]) OR (Neuropathies, Diabetic Autonomic[Title/Abstract]) OR (Neuropathy, Diabetic Autonomic[Title/Abstract]) OR (Diabetic Neuralgia[Title/Abstract]) OR (Diabetic Neuralgias[Title/Abstract]) OR (Neuralgias, Diabetic[Title/Abstract]) OR (Diabetic Neuropathy, Painful[Title/Abstract]) OR (Diabetic Neuropathies, Painful[Title/Abstract]) OR (Neuropathies, Painful Diabetic[Title/Abstract]) OR (Neuropathy, Painful Diabetic[Title/Abstract]) OR (Painful Diabetic Neuropathies[Title/Abstract]) OR (Painful Diabetic Neuropathy[Title/Abstract]) OR (Neuralgia, Diabetic[Title/Abstract]) OR (Symmetric Diabetic Proximal Motor Neuropathy[Title/Abstract]) OR (Asymmetric Diabetic Proximal Motor Neuropathy[Title/Abstract]) OR (Diabetic Asymmetric Polyneuropathy[Title/Abstract]) OR (Asymmetric Polyneuropathies, Diabetic[Title/Abstract]) OR (Asymmetric Polyneuropathy, Diabetic[Title/Abstract]) OR (Diabetic Asymmetric Polyneuropathies[Title/Abstract]) OR (Polyneuropathies, Diabetic Asymmetric[Title/Abstract]) OR (Polyneuropathy, Diabetic Asymmetric[Title/Abstract]) OR (Diabetic Mononeuropathy[Title/Abstract]) OR (Diabetic Mononeuropathies[Title/Abstract]) OR (Mononeuropathies, Diabetic[Title/Abstract]) OR (Mononeuropathy, Diabetic[Title/Abstract]) OR (Diabetic Mononeuropathy Simplex[Title/Abstract]) OR (Diabetic Mononeuropathy Simplices[Title/Abstract]) OR (Mononeuropathy Simplex, Diabetic[Title/Abstract]) OR (Mononeuropathy Simplices, Diabetic[Title/Abstract]) OR (Simplex, Diabetic Mononeuropathy[Title/Abstract]) OR (Simplices, Diabetic Mononeuropathy[Title/Abstract]) OR (Diabetic Amyotrophy[Title/Abstract]) OR (Amyotrophies, Diabetic[Title/Abstract]) OR (Amyotrophy, Diabetic[Title/Abstract]) OR (Diabetic Amyotrophies[Title/Abstract]) OR (Diabetic Polyneuropathy[Title/Abstract]) OR (Diabetic Polyneuropathies[Title/Abstract]) OR (Polyneuropathies, Diabetic[Title/Abstract]) OR (Polyneuropathy, Diabetic[Title/Abstract]) OR (Diabetic peripheral neuropathy[Title/Abstract])

#3 #1 OR #2

#4 Stem Cells [MeSH Terms]

#5 (Cell, Stem[Title/Abstract]) OR (Cells, Stem[Title/Abstract]) OR (Stem Cell[Title/Abstract]) OR (Progenitor Cells[Title/Abstract]) OR (Cell, Progenitor[Title/Abstract]) OR (Cells, Progenitor[Title/Abstract]) OR (Progenitor Cell[Title/Abstract]) OR (Mother Cells[Title/Abstract]) OR (Cell, Mother[Title/Abstract]) OR (Cells, Mother[Title/Abstract]) OR (Mother Cell[Title/Abstract]) OR (Colony-Forming Unit[Title/Abstract]) OR (Colony Forming Unit[Title/Abstract]) OR (Colony-Forming Units[Title/Abstract]) OR (Colony Forming Units[Title/Abstract])

#6 #4 OR #5

#7 #3 AND #6

**Cochran library:**

#1 MeSH descriptor: [Diabetic Neuropathies] explode all trees

#2 (Diabetic Neuropathy OR Neuropathies, Diabetic OR Neuropathy, Diabetic OR Diabetic Autonomic Neuropathy OR Autonomic Neuropathies, Diabetic OR Autonomic Neuropathy, Diabetic OR Diabetic Autonomic Neuropathies OR Neuropathies, Diabetic Autonomic OR Neuropathy, Diabetic Autonomic OR Diabetic Neuralgia OR Diabetic Neuralgias OR Neuralgias, Diabetic OR Diabetic Neuropathy, Painful OR Diabetic Neuropathies, Painful OR Neuropathies, Painful Diabetic OR Neuropathy, Painful Diabetic OR Painful Diabetic Neuropathies OR Painful Diabetic Neuropathy OR Neuralgia, Diabetic OR Symmetric Diabetic Proximal Motor Neuropathy OR Asymmetric Diabetic Proximal Motor Neuropathy OR Diabetic Asymmetric Polyneuropathy OR Asymmetric Polyneuropathies, Diabetic OR Asymmetric Polyneuropathy, Diabetic OR Diabetic Asymmetric Polyneuropathies OR Polyneuropathies, Diabetic Asymmetric OR Polyneuropathy, Diabetic Asymmetric OR Diabetic Mononeuropathy OR Diabetic Mononeuropathies OR Mononeuropathies, Diabetic OR Mononeuropathy, Diabetic OR Diabetic Mononeuropathy Simplex OR Diabetic Mononeuropathy Simplices OR Mononeuropathy Simplex, Diabetic OR Mononeuropathy Simplices, Diabetic OR Simplex, Diabetic Mononeuropathy OR Simplices, Diabetic Mononeuropathy OR Diabetic Amyotrophy OR Amyotrophies, Diabetic OR Amyotrophy, Diabetic OR Diabetic Amyotrophies OR Diabetic Polyneuropathy OR Diabetic Polyneuropathies OR Polyneuropathies, Diabetic OR Polyneuropathy, Diabetic OR Diabetic peripheral neuropathy) :ti,ab,kw

#3 #1 OR #2

#4 MeSH descriptor: [Stem Cells] explode all trees

#5 (Cell, Stem OR Cells, Stem OR Stem Cell OR Progenitor Cells OR Cell, Progenitor OR Cells, Progenitor OR Progenitor Cell OR Mother Cells OR Cell, Mother OR Cells, Mother OR Mother Cell OR Colony-Forming Unit OR Colony Forming Unit OR Colony-Forming Units OR Colony Forming Units) :ti,ab,kw

#6 #4 OR #5

#7 #3 AND #6

**EMBASE**

#1 ' Diabetic Neuropathies '/exp

#2 ‘Diabetic Neuropathy’:ab,ti OR ‘Neuropathies, Diabetic’:ab,ti OR ‘Neuropathy, Diabetic’:ab,ti OR ‘Diabetic Autonomic Neuropathy’:ab,ti OR ‘Autonomic Neuropathies, Diabetic’:ab,ti OR ‘Autonomic Neuropathy, Diabetic’:ab,ti OR ‘Diabetic Autonomic Neuropathies’:ab,ti OR ‘Neuropathies, Diabetic Autonomic’:ab,ti OR ‘Neuropathy, Diabetic Autonomic’:ab,ti OR ‘Diabetic Neuralgia’:ab,ti OR ‘Diabetic Neuralgias’:ab,ti OR ‘Neuralgias, Diabetic’:ab,ti OR ‘Diabetic Neuropathy, Painful’:ab,ti OR ‘Diabetic Neuropathies, Painful’:ab,ti OR ‘Neuropathies, Painful Diabetic’:ab,ti OR ‘Neuropathy, Painful Diabetic’:ab,ti OR ‘Painful Diabetic Neuropathies’:ab,ti OR ‘Painful Diabetic Neuropathy’:ab,ti OR ‘Neuralgia, Diabetic’:ab,ti OR ‘Symmetric Diabetic Proximal Motor Neuropathy’:ab,ti OR ‘Asymmetric Diabetic Proximal Motor Neuropathy’:ab,ti OR ‘Diabetic Asymmetric Polyneuropathy’:ab,ti OR ‘Asymmetric Polyneuropathies, Diabetic’:ab,ti OR ‘Asymmetric Polyneuropathy, Diabetic’:ab,ti OR ‘Diabetic Asymmetric Polyneuropathies’:ab,ti OR ‘Polyneuropathies, Diabetic Asymmetric’:ab,ti OR ‘Polyneuropathy, Diabetic Asymmetric’:ab,ti OR ‘Diabetic Mononeuropathy’:ab,ti OR ‘Diabetic Mononeuropathies’:ab,ti OR ‘Mononeuropathies, Diabetic’:ab,ti OR ‘Mononeuropathy, Diabetic’:ab,ti OR ‘Diabetic Mononeuropathy Simplex’:ab,ti OR ‘Diabetic Mononeuropathy Simplices’:ab,ti OR ‘Mononeuropathy Simplex, Diabetic’:ab,ti OR ‘Mononeuropathy Simplices, Diabetic’:ab,ti OR ‘Simplex, Diabetic Mononeuropathy’:ab,ti OR ‘Simplices, Diabetic Mononeuropathy’:ab,ti OR ‘Diabetic Amyotrophy’:ab,ti OR ‘Amyotrophies, Diabetic’:ab,ti OR ‘Amyotrophy, Diabetic’:ab,ti OR ‘Diabetic Amyotrophies’:ab,ti OR ‘Diabetic Polyneuropathy’:ab,ti OR ‘Diabetic Polyneuropathies’:ab,ti OR ‘Polyneuropathies, Diabetic’:ab,ti OR ‘Polyneuropathy, Diabetic’:ab,ti OR ‘Diabetic peripheral neuropathy’:ab,ti

#3 #1 OR #2

#4 ‘Stem Cells’/exp

#5 ‘Cell, Stem’:ab,ti OR ‘Cells, Stem’:ab,ti OR ‘Stem Cell’:ab,ti OR ‘Progenitor Cells’:ab,ti OR ‘Cell, Progenitor’:ab,ti OR ‘Cells, Progenitor’:ab,ti OR ‘Progenitor Cell’:ab,ti OR ‘Mother Cells’:ab,ti OR ‘Cell, Mother’:ab,ti OR ‘Cells, Mother’:ab,ti OR ‘Mother Cell’:ab,ti OR ‘Colony-Forming Unit’:ab,ti OR ‘Colony Forming Unit’:ab,ti OR ‘Colony-Forming Units’:ab,ti OR ‘Colony Forming Units’:ab,ti

#6 #4 OR #5

#7 #3 AND #6

**Web of science**

#1 TS=(Stem Cells OR Cell, Stem OR Cells, Stem OR Stem Cell OR Progenitor Cells OR Cell, Progenitor OR Cells, Progenitor OR Progenitor Cell OR Mother Cells OR Cell, Mother OR Cells, Mother OR Mother Cell OR Colony-Forming Unit OR Colony Forming Unit OR Colony-Forming Units OR Colony Forming Units)

#2 TS=(Diabetic Neuropathies OR Diabetic Neuropathy OR Neuropathies, Diabetic OR Neuropathy, Diabetic OR Diabetic Autonomic Neuropathy OR Autonomic Neuropathies, Diabetic) OR (Autonomic Neuropathy, Diabetic OR Diabetic Autonomic Neuropathies OR Neuropathies, Diabetic Autonomic OR Neuropathy, Diabetic Autonomic OR Diabetic Neuralgia OR Diabetic Neuralgias OR Neuralgias, Diabetic OR Diabetic Neuropathy, Painful OR Diabetic Neuropathies, Painful OR Neuropathies, Painful Diabetic OR Neuropathy, Painful Diabetic OR Painful Diabetic Neuropathies OR Painful Diabetic Neuropathy OR Neuralgia, Diabetic OR Symmetric Diabetic Proximal Motor Neuropathy OR Asymmetric Diabetic Proximal Motor Neuropathy OR Diabetic Asymmetric Polyneuropathy OR Asymmetric Polyneuropathies, Diabetic OR Asymmetric Polyneuropathy, Diabetic OR Diabetic Asymmetric Polyneuropathies OR Polyneuropathies, Diabetic Asymmetric OR Polyneuropathy, Diabetic Asymmetric OR Diabetic Mononeuropathy OR Diabetic Mononeuropathies OR Mononeuropathies, Diabetic OR Mononeuropathy, Diabetic OR Diabetic Mononeuropathy Simplex OR Diabetic Mononeuropathy Simplices OR Mononeuropathy Simplex, Diabetic OR Mononeuropathy Simplices, Diabetic OR Simplex, Diabetic Mononeuropathy OR Simplices, Diabetic Mononeuropathy OR Diabetic Amyotrophy OR Amyotrophies, Diabetic OR Amyotrophy, Diabetic OR Diabetic Amyotrophies OR Diabetic Polyneuropathy OR Diabetic Polyneuropathies OR Polyneuropathies, Diabetic OR Polyneuropathy, Diabetic OR Diabetic peripheral neuropathy)

#3 #1 AND #2
